# Evaluation of *in vitro* antiviral activity of SARS-CoV-2 M^pro^ inhibitor pomotrelvir and cross-resistance to nirmatrelvir resistance substitutions

**DOI:** 10.1128/aac.00840-23

**Published:** 2023-10-06

**Authors:** Xiao Tong, Walter Keung, Lee D. Arnold, Laura J. Stevens, Andrea J. Pruijssers, Seunghyi Kook, Uri Lopatin, Mark Denison, Ann D. Kwong

**Affiliations:** 1 Pardes Biosciences, Inc., Carlsbad, California, USA; 2 Vanderbilt University Medical Center, Nashville, Tennessee, USA; IrsiCaixa Institut de Recerca de la Sida, Badalona, Barcelona, Spain

**Keywords:** pomotrelvir, PBI-0451, Mpro inhibitor, SARS-CoV-2, coronavirus, antiviral resistance

## Abstract

The unprecedented scale of the COVID-19 pandemic and the rapid evolution of SARS-CoV-2 variants underscore the need for broadly active inhibitors with a high barrier to resistance. The coronavirus main protease (M^pro^) is an essential cysteine protease required for viral polyprotein processing and is highly conserved across human coronaviruses. Pomotrelvir is a novel M^pro^ inhibitor that has recently completed a phase 2 clinical trial. In this report, we demonstrated that pomotrelvir is a potent competitive inhibitor of SARS-CoV-2 M^pro^ with high selectivity against human proteases. In the enzyme assay, pomotrelvir is also active against M^pro^ proteins derived from human coronaviruses CoV-229E, CoV-OC43, CoV-HKU1, CoV-NL63, MERS, and SARS-CoV. In cell-based SARS-CoV-2 replicon and SARS-CoV-2 infection assays, pomotrelvir has shown potent inhibitory activity and is broadly active against SARS-CoV-2 clinical isolates including Omicron variants. Many resistance substitutions of the M^pro^ inhibitor nirmatrelvir confer cross-resistance to pomotrelvir, consistent with the finding from our enzymatic analysis that pomotrelvir and nirmatrelvir compete for the same binding site. In a SARS-CoV-2 infection assay, pomotrelvir is additive when combined with remdesivir or molnupiravir, two nucleoside analogs targeting viral RNA synthesis. In conclusion, our results from the *in vitro* characterization of pomotrelvir antiviral activity support its further clinical development as an alternative COVID-19 therapeutic option.

## INTRODUCTION

Coronaviruses (CoVs) are enveloped, positive-sense, single-stranded RNA viruses; seven of which are known to infect humans (SARS-CoV-2, SARS-CoV, MERS, CoV-229E, CoV-OC43, CoV-NL63, and CoV-HKU1) (1, 2). SARS-CoV-2 was discovered in late 2019 and is responsible for the coronavirus disease 2019 (COVID-19) pandemic. It is the latest addition to human CoVs and represents the third novel CoV (in addition to SARS-CoV and MERS) to make a zoonotic transfer in the last 20 y and result in severe human diseases (3). To date, over 670 million people have been diagnosed with COVID-19 and the death toll has exceeded 6.8 million patients worldwide (4). Symptoms of COVID-19 include extreme fatigue and dyspnea that can be short lived or prolonged (e.g., “long-haulers” and “Long COVID”), with some cases developing into acute respiratory distress syndrome, with or without pneumonia, resulting in hospitalization and sometimes death, despite aggressive supportive care (5 – 7). While all persons are at risk for acquiring COVID-19, there are a number of high-risk populations, including the elderly, those living in close contact with others (e.g., long-term care facilities), and those with chronic preexisting medical conditions such as chronic immune suppression and cardiovascular, metabolic (i.e., diabetes), and lung diseases (https://www.cdc.gov/coronavirus/2019-ncov/hcp/clinical-care/underlyingconditions.html). In addition, the other four human CoVs (229E, OC43, NL63, and HKU1) are believed to result in 5%–30% of “common colds,” for which therapeutic intervention is generally limited to over-the-counter symptomatic relief (8).

Following binding to a cell, CoVs release their positive-stranded RNA genome into the host cell cytoplasm, where the host ribosome transcribes the viral RNA into two large polyproteins that provide the proteins required for viral replication. The main viral protease (M^pro^) is essential for viral replication by processing the majority (11 of 16 cleavages) of the viral polyprotein into individual functional proteins. The liberated mature proteins are subsequently assembled into the replication machinery and participate in the transcription of new viral RNA which are packaged into virions to produce infectious CoV progeny virus (1). The CoV M^pro^ protein, also known as 3-chymotrypsin like protease, is a cysteine protease composed of about 300 amino acids embedded in the polyproteins as the nsp5 domain. It has a Cys-His catalytic dyad (Cys145 and His41), in which Cys145 acts as a nucleophile and His41 functions as the acid base in the proteolytic process. M^pro^ exclusively cleaves polypeptides after a Gln residue, and no known human protease displays the same cleavage specificity, which favors the discovery of M^pro^ inhibitors with high selectivity and low toxicity to host cells. Therefore, M^pro^ has been an attractive drug target in the development of SARS-CoV-2 inhibitors (9 – 11).

Two classes of direct-acting antiviral (DAA) drugs have received worldwide or US regulatory Emergency Use Authorization or New Drug Authorization (NDA) approval to treat SARS-CoV-2 infections in high-risk patients: molnupiravir and remdesivir are inhibitors targeting the viral RNA-dependent RNA polymerase (RdRp) (12); in the class of M^pro^ inhibitors, nirmatrelvir (co-administered with ritonavir [RTV]) is the only SARS-CoV-2 M^pro^ inhibitor with NDA approval in the United States for treating high-risk patients (https://www.fda.gov/news-events/press-announcements/fda-approves-first-oral-antiviral-treatment-covid-19-adults). A second M^pro^ inhibitor (ensitrelvir) has received emergency regulatory approval for the treatment of high-risk COVID-19 patients only in Japan (12). Even though tremendous progress has been made in the treatment of COVID-19 in the past couple of years, there is still a need for safe and efficacious oral drugs to overcome limitations of current DAA therapies. For example, molnupiravir inhibits viral replication by increasing the frequency of viral mutagenesis and it has the potential to cause embryo-fetal toxicity and affect bone and cartilage growth, which significantly restricts its use in women of childbearing age and their male partners and children, respectively (13). The viral polymerase inhibitor remdesivir is only available for IV administration in a setting with the resources to manage severe hypersensitivity reactions, including anaphylaxis (https://www.fda.gov/media/143189/download). The M^pro^ inhibitor nirmatrelvir requires co-administration with RTV, which introduces significant drug-drug interaction considerations in high-risk patients who are also on medications metabolized by the cytochrome P450 3A pathway (14). Furthermore, no SARS-CoV-2 antivirals have been approved for the vast majority of patients who are not at high risk for serious COVID-19 symptoms but who nevertheless suffer from acute diseases and may be at risk for developing “Long COVID.” To address this need, pomotrelvir (PBI-0451) has been developed as a non-RTV-containing M^pro^ regimen to treat both standard and high-risk patients and to prevent or reduce transmission. It has completed phase 1 studies in healthy human patients (NCT05011812) and phase 2 studies in otherwise healthy COVID-19 patients not at risk of developing serious diseases (NCT05543707). In this report, we describe pomotrelvir potency and mechanism of action, broad activity against SARS-CoV-2 clinical isolates, combination studies with other SARS-CoV-2 inhibitors, and cross-resistance profile compared with nirmatrelvir.

## RESULTS

### Pomotrelvir is a potent competitive inhibitor of SARS-CoV-2 M^pro^

**Fig 1:**
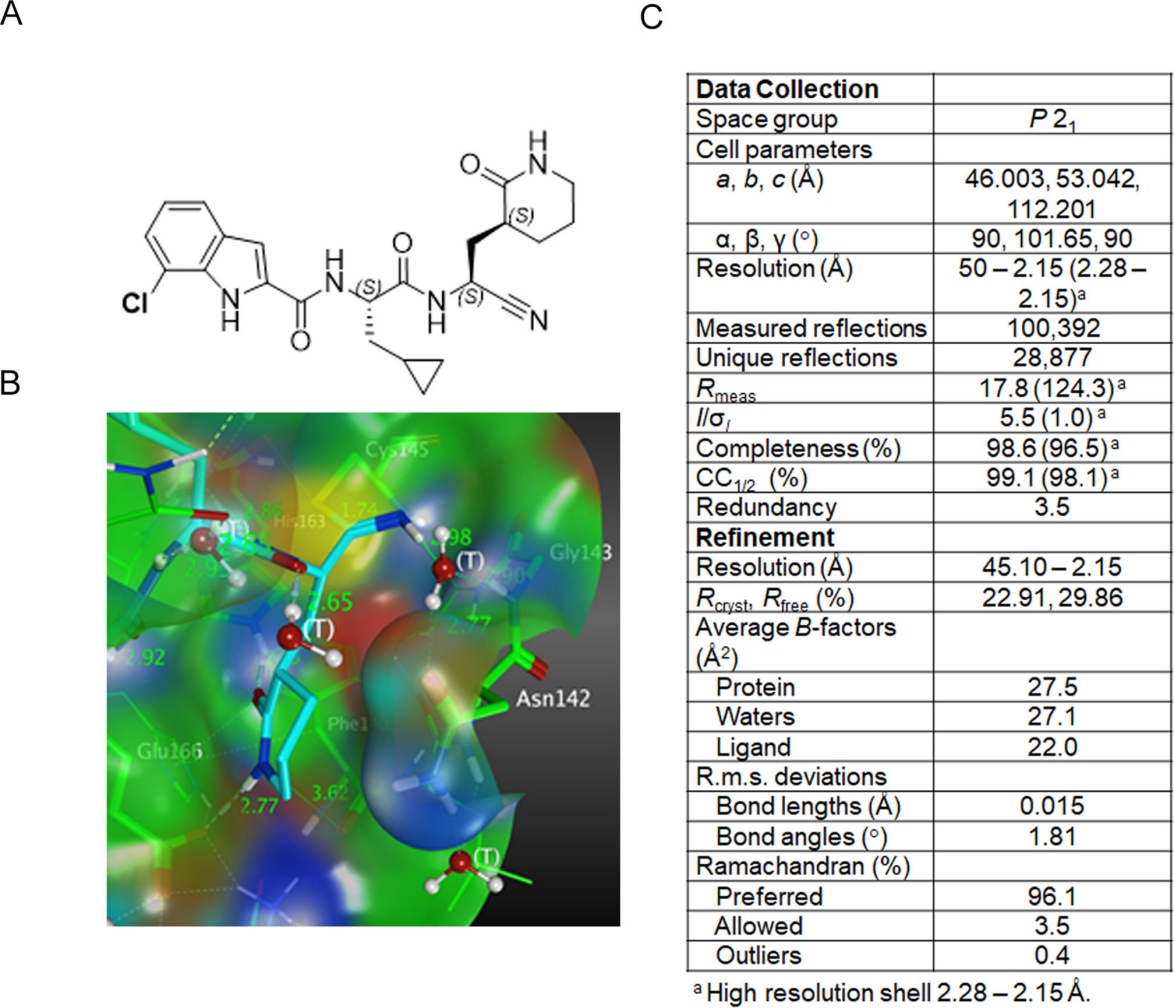
Structural analysis of pomotrelvir. (A) Chemical structure of pomotrelvir. (**B)** Crystal structure of pomotrelvir bound to SARS-CoV-2 M^pro^. (**C) **X-ray diffraction data collection and refinement statistics.

**Fig 2:**
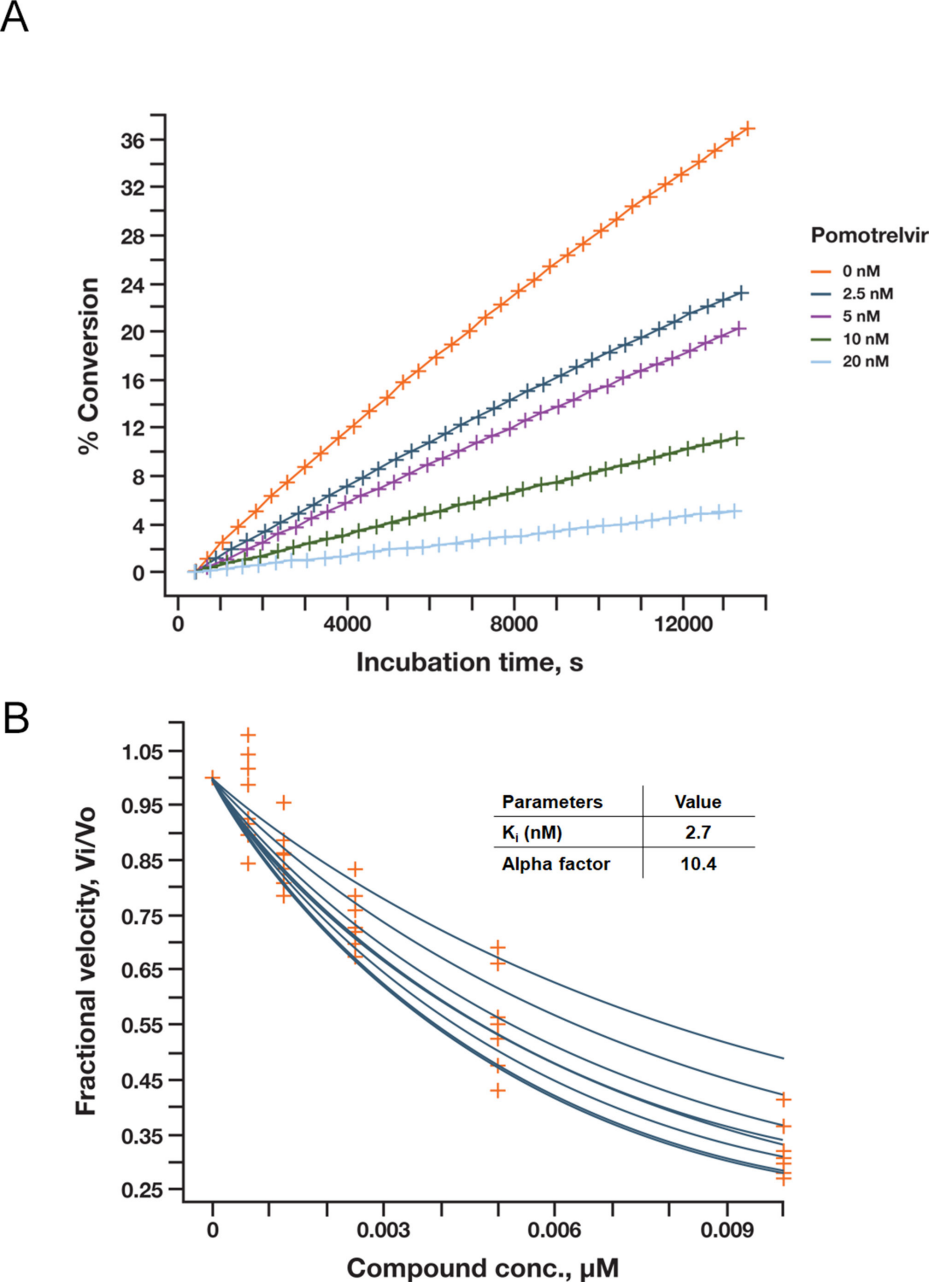
Characterization of pomotrelvir (PBI-0451) inhibition of SARS-CoV-2 M^pro^ in enzyme assays. (A) Pomotrelvir was tested in the presence of 3 nM SARS-CoV-2 M^pro^ and 1 µM of substrate peptide (FAMTSAVLQSGFRK-NH2); cleavage was measured by microfluidic electrophoresis using Caliper’s LabChip 3000. Progress curve was generated by plotting conversion% of substrate over incubation time. (**B**) The *K*_i_ for pomotrelvir was obtained by a global fit with the Morrison correction (with 2.3–300 μM substrate); an alpha factor of 10.4 (>>1) was consistent with a competitive inhibition model.

**Fig 3:**
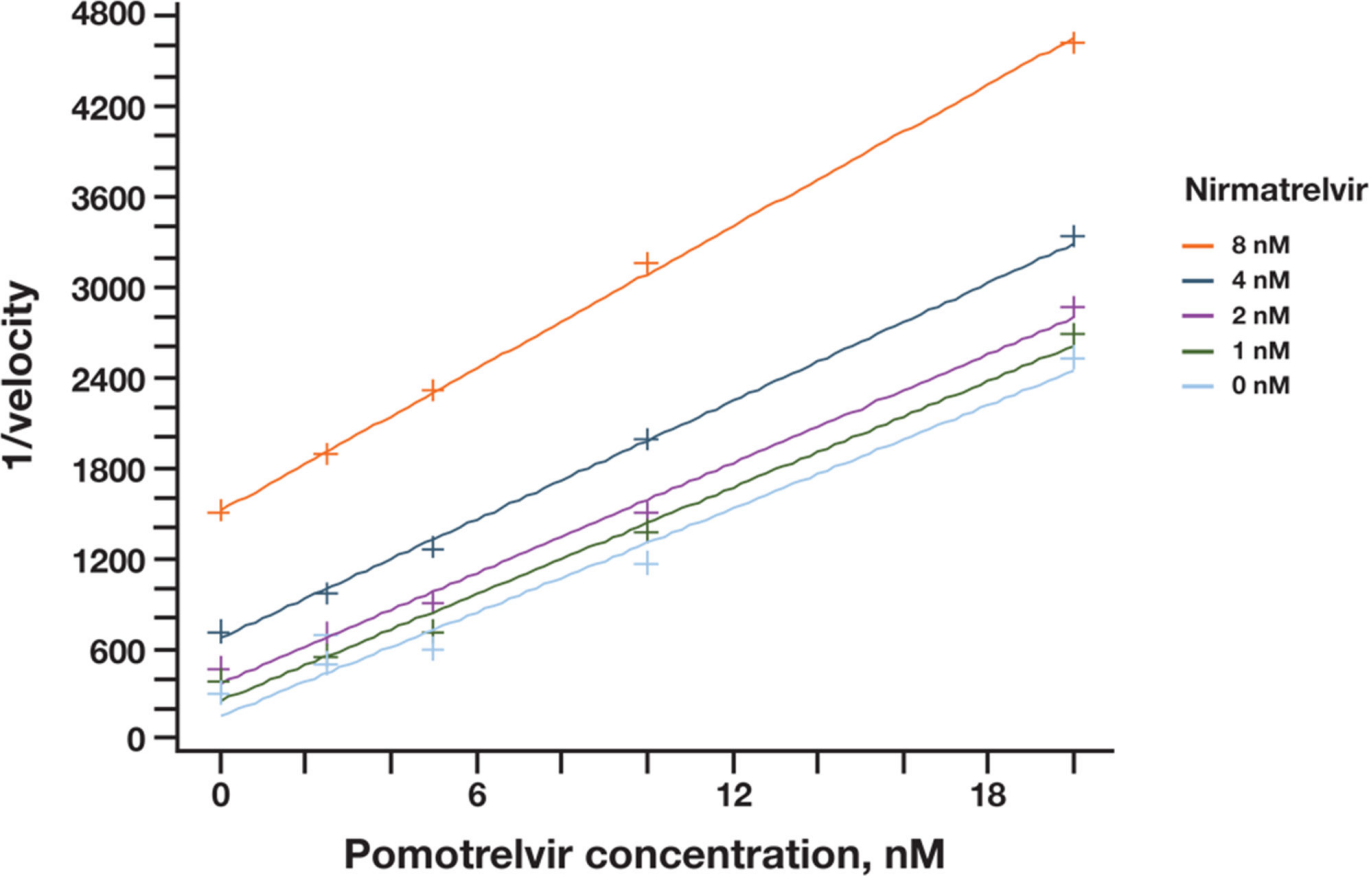
Binding exclusivity study of pomotrelvir (PBI-0451) and nirmatrelvir. The initial velocity was determined in the presence of pomotrelvir (0–20 nM) and nirmatrelvir (0–8 nM). The binding mode was assessed by the Yonetani–Theorell equation. The binding exclusivity coefficient (*γ*) was >>1 as shown by the near-parallel lines under various nirmatrelvir concentrations, demonstrating that the two inhibitors share the same binding site.

Pomotrelvir (Fig. 1A) was discovered by Pardes Biosciences through structure-based drug design using crystal structures and models of human CoV M^pro^ proteins alone and in co-complex with M^pro^ inhibitors. The co-crystal structure of pomotrelvir bound to SARS-CoV-2 M^pro^ was solved at a resolution of 2.15 Å (Fig. 1B and C). A covalent adduct was observed between the nitrile warhead and Cys145 as evidenced by the resultant thioimidate with a C-S distance of 1.79 Å. The result is similar to what has been reported with the M^pro^ inhibitor nirmatrelvir (PF-07321332) in which the nitrile substituent forms a reversible covalent thioimidate adduct with the catalytic Cys145 (15). The inhibitory activity of pomotrelvir was first evaluated in a SARS-CoV-2 M^pro^ kinetic assay using highly sensitive microfluidics capillary electrophoresis to detect cleaved peptide substrate. As shown in Fig. 2A, the progress curve was linear as a function of time. The inhibition constant (*K*_i_) was calculated to be 2.7 nM; the alpha factor was 10.4 (>>1), suggestive of the competitive inhibition model (Fig. 2B). An enzyme exclusivity binding study (Fig. 3) between pomotrelvir and nirmatrelvir showed that the binding exclusivity coefficient (*γ*) approached ∞ when combinations of both inhibitors (1–8 nM) were present in the assay, demonstrating that pomotrelvir and nirmatrelvir compete for the same active site and their binding to the M^pro^ enzyme is mutually exclusive.

The inhibitory activity of pomotrelvir against M^pro^ enzymes derived from other human CoVs was evaluated in an endpoint IC50 assay. Pomotrelvir inhibited wild-type (WT) SARS-CoV-2 M^pro^ with an IC50 of 24 nM. Similar activity (IC50 of 34 nM) was observed with the P132H variant, which is commonly observed in Omicron isolates and has been shown to have no effect on nirmatrelvir inhibition (16) (Table 1). Moreover, pomotrelvir showed broad-spectrum activity against M^pro^ enzymes from other pathogenic and common cold CoV strains with IC50 ranging from 61 to 379 nM (Table 1).

**TABLE 1:** Pomotrelvir inhibition of M^pro^ from SARS-CoV2 and other human CoVs

M^pro^ source	^a^Mean IC50 (nM, SD)
SARS-CoV2 WT	24 (5)
SARS-CoV2_P132H (Omicron)	34 (10)
CoV-229E	141 (38)
CoV-OC43	158 (33)
CoV-HKU1	61 (29)
CoV-MERS	379 (106)
CoV-NL63	206 (84)
CoV-SARS	45 (17)

^a^ Mean from 4 to 8 experiments.

### Pomotrelvir exhibits high selectivity against host proteases

Potential off-target activity of pomotrelvir against host serine and cysteine proteases was evaluated in the microfluidic assay with relevant peptidyl substrates. As described above, pomotrelvir showed a *K*_i_ of 2.7 nM against the SARS-CoV-2 M^pro^. For human cysteine and serine proteases caspase-2, chymotrypsin C, elastase, and thrombin, no inhibitory activity was observed at 100 µM pomotrelvir, resulting in >37,000-fold selectivity over the *K*_i_ of SARS-CoV-2 M^pro^. For caspase 3, calpain1, cathepsin D, and dipeptidyl peptidase IV, no inhibitory activity was observed at 30 µM pomotrelvir, resulting in >11,000-fold selectivity. Pomotrelvir showed 165-, 107-, 470-, and 2,740-fold selectivity against cathepsin S (*K*_i_ = 0.445 µM), cathepsin K (*K*_i_ = 0.289 µM), cathepsin B (*K*_i_ = 1.27 µM), and cathepsin L (*K*_i_ = 7.4 µM), respectively. These results demonstrated that pomotrelvir is highly selective against host serine and cysteine proteases.

### Pomotrelvir inhibits SARS-CoV-2 in cell-based assays

The inhibitory activity of pomotrelvir against SARS-CoV-2 was assessed using three cell-based systems: (i) Pluripotent induced stem cell-derived human lung alveolar type 2 (iPS-AT2) cells were infected with passage 5 of SARS-CoV-2 WA-1 (Washington State isolation of the original Wuhan strain). Viral replication was quantified using a plaque assay to measure reduction in infectious viral titer and a RT-PCR assay to measure reduction in viral RNA copy number. (ii) A549-hACE2 cells overexpressing the human ACE2 receptor for SARS-CoV-2 were infected with SARS-CoV-2 NLuc, generated by inserting the nanoluciferase gene into the SARS-CoV-2 WA1 strain which allows viral replication to be quantified by measuring nanoluciferase activity. (iii) Huh7 cells were transfected with the SARS-CoV-2-EGFP replicon RNA; the viral replication was measured by the expression of the EGFP reporter. As summarized in Table 2, pomotrelvir showed similar activity in all three assay systems. The mean EC50 values of 32 nM (by plaque assay) and 36 nM (by qRT-PCR) in the SARS CoV-2 assay in iPS-AT2 cells and 23 nM in the SARS-CoV-2 NLuc assay were in close concordance with each other and were also consistent with the mean EC50 of 27 nM obtained with the SARS-CoV-2-EGFP replicon assay.

**TABLE 2:** Pomotrelvir antiviral activity and cytotoxicity in cell-based assays^*b*^

Virus	Cell line	Assay	^*a*^Mean EC50 (nM, SD)	^*a*^Mean EC90 (nM, SD)	CC50 (μM)	SI
SARS-CoV-2 WA1	iPS-AT2	Plaque assay	32 (29)	106 (103)	>2	>63
SARS-CoV-2 WA1	iPS-AT2	RT-PCR assay	36 (22)	67 (41)	>2	>56
SARS-CoV-2 WA1 NLuc	A549-ACE2	Nanoluciferase reporter	23 (17)	113.5 (93)	>10	>435
SARS-CoV-2 Wuhan replicon	Huh7	EGFP reporter	27 (10)	150 (20)	>10	>370
CoV-229E	MRC5	CPE assay	180 (70)	410 (220)	>90	>500
CoV-OC43	Huh7	CPE assay	380 (100)	490 (250)	>90	>237

^*a*^ Mean from 4 to 12 experiments.

^*b*^ SI, selectivity index is calculated as CC50/EC50.

Additionally, pomotrelvir antiviral activity was assessed against CoV-229E and CoV-OC43. Pomotrelvir was active in the CoV 229E and CoV-OC43 infectious virus CPE assays, with mean EC50 values of 180 nM and 380 nM, respectively (Table 2). Cytotoxicity was measured in parallel by determining cell viability in uninfected cells treated with pomotrelvir. In all cell lines tested, the CC50 for pomotrelvir was above the highest concentration tested (up to >90 µM), with a selectivity index ranging from >56 to >500 (Table 2).

The effect of human serum on pomotrelvir antiviral activity was evaluated in the SARS-CoV-2 NLuc assay. As shown in Table 3, the addition of 5%, 10%, and 20% human sera (in the presence of 10% fetal bovine serum [FBS]) resulted in a concentration-dependent increase in EC50 and EC90. Extrapolating to 100% human serum by linear regression resulted in estimated shifts of 22-fold for EC50 and 13-fold for EC90.

**TABLE 3:** Effect of human serum on pomotrelvir antiviral activity in the SARS-CoV-2 NLuc assay

Human serum concentration	EC50 (nM)	Fold	EC90 (nM)	Fold
0% human serum + (10% FBS)	9.9	−	35	−
5% human serum + (10% FBS)	16.6	1.7	96.2	2.8
10% human serum + (10% FBS)	23.8	2.4	118.9	3.4
20% human serum + (10% FBS)	53	5.3	125.9	3.6

The activity of pomotrelvir against clinical isolates was evaluated in A549-AT cells (A549 cells overexpressing ACE2 and TMPRSS2). After infection of A549-AT cells, immunostaining of the viral nucleoprotein was used to monitor infection and determine levels of viral replication. Table 4 summarizes the results of 11 SARS-CoV-2 clinical isolates representing the D614G B.1, Alpha, Delta, Epsilon, Mu, and Omicron variants. Pomotrelvir was fully active across all clinical isolates including five Omicron variants; the fold change over the ancestral strain (D614G B.1) was 0.5- to 2.5-fold for EC50 and 0.3- to 2.3-fold for EC90. Sequence analysis of SARS-CoV-2 isolates discussed above showed that the M^pro^ region and the 11 M^pro^ cleavage sites were well conserved; the only substitution of note was the P132H change, which was observed in all Omicron isolates used in our study (data not shown).

**TABLE 4:** Pomotrelvir antiviral activity against SARS-CoV-2 clinical variants in A549-AT cells

SARS-CoV-2 variant	^a^EC50		^a^EC90	
	Mean (nM, SD)	Mean fold	Mean (nM, SD)	Mean fold
D614G B.1 (ancestral)	151 (72)	−	547 (377)	−
Alpha	95 (22)	0.6	144 (9)	0.3
Epsilon	195 (14)	1.3	777 (65)	1.4
Delta	251 (42)	1.7	425 (110)	0.8
AY.20 Delta	374 (19)	2.5	672 (55)	1.2
Mu	73 (15)	0.5	195 (104)	0.4
BA.1.1 Omicron	94 (4)	0.6	213 (23)	0.4
BA.2 Omicron	139 (63)	0.9	668 (486)	1.2
BA.2.12.1 Omicron	60 (2)	0.4	171 (10)	0.3
BA.4.1 Omicron	244 (145)	1.6	1120 (768)	2.0
BA.5.2.1 Omicron	256 (174)	1.7	1270 (1070)	2.3

^a^ Mean from 2 to 6 experiments.

### Cross-resistance to pomotrelvir is observed with certain nirmatrelvir resistance substitutions

Nirmatrelvir is one of the first M^pro^ inhibitors developed for SARS-CoV-2 (15), and resistance substitutions in the M^pro^ sequence have been reported (14, 17 – 19). Given the finding that pomotrelvir and nirmatrelvir compete for the same enzyme binding site, we investigated whether pomotrelvir would be similarly affected by nirmatrelvir resistance substitutions. We first evaluated the effect of substitutions on M^pro^ enzymatic activity. As shown in Table 5, the majority of the 37 mutant M^pro^ enzymes containing single or multiple substitutions had similar or better catalytic efficiency (*k*_cat_/*K*_m_) compared with the WT protease. The few exceptions were enzymes containing substitutions Y54A, E166A, E166V, L167F, and H172Y (as single mutations or in combination with other substitutions), with catalytic efficiency ~5- to ~10-fold lower than that of the WT enzyme.

**TABLE 5:** Enzyme activity and pomotrelvir susceptibility of mutant M^pro^ proteins containing nirmatrelvir resistance substitutions

^a^Enzyme	*K*_m_ (μM)	*K*_cat_ (s-1)	*k*_cat_/*K*_m_ × 10^6^ (M-1 s-1)	^b^PBI-0451		^b^GC376	
				Mean IC50 (μM)	Mean fold	IC50 (μM)	Fold
WT	14	41	3	0.026	–	0.02	–
A173V + T304I	11	13	1.2	0.16	6.7	0.10	5.5
A260V	8	48	6	0.024	0.9	0.02	1.0
D248E	9	37	4.3	0.025	1	0.03	1.3
E166A	13	2.7	0.2	0.95	36	0.32	14
E166V	15	4.8	0.3	1.2	51	0.66	35
F140A	12	24	2	0.37	14	0.15	6.8
F140L + A173V	11	41	3.9	0.23	10	0.14	7.5
G15S	9	29	3.2	0.027	1	0.02	0.9
H164N	10	26	2.7	0.064	2.4	0.08	3.5
H172Y	25	4.5	0.2	1.94	71	0.41	18
H172Y + P252L	12	9.8	0.8	1.5	62	0.28	15
K90R	11	33	2.9	0.023	0.9	0.01	0.6
L167F	11	5.5	0.5	0.34	13	0.13	5.6
L50F	7	64	8.6	0.03	1.1	0.02	1.1
L50F + A173V	7	28	4.1	0.082	3.5	0.06	3.4
L50F + E166A	21	10	0.5	0.87	35	0.27	12
L50F + E166A + L167F	14	5.4	0.4	5.0	188	0.76	34
L50F + E166V	19	6.9	0.4	1.0	45	0.43	23
L50F + F140L + L167F + T304I	14	26	1.9	0.9	40	0.17	8.9
L50F + T304I	14	120	8.5	0.065	2.8	0.06	3.3
Q189K	13	14	1.1	0.071	2.7	0.05	2.1
S144A	9	26	2.9	0.18	6.7	0.12	5.2
T135I	12	51	4.3	0.047	1.8	0.02	0.8
T135I + T304I	7	44	6	0.087	3.7	0.07	3.8
T21I	10	82	8	0.027	1.0	0.02	1.0
T21I + A173V	8	37	4.7	0.11	4.7	0.08	4.2
T21I + A173V + T304I	15	35	2.3	0.14	6.1	0.09	5.0
T21I + C160F + A173V+ V186A + T304I	14	38	2.7	0.13	5.7	0.09	4.9
T21I + E166V	13	2.7	0.2	1.24	55	0.59	31
T21I + L50F	7	80	12.2	0.021	0.8	0.02	0.9
T21I + L50F + A193*P* + S301P	7	25	3.4	0.11	4.9	0.07	3.6
T21I + S144A	15	55	3.7	0.31	13	0.12	6.4
T21I + S144A + T304I	11	13	1.3	0.52	23	0.19	10
T21I + T304I	9	75	8.4	0.071	3.1	0.05	2.6
Y54A	11	2.3	0.2	0.15	5.6	0.16	7.0

^a^ Mutant enzymes are listed in alphabetic order.

^b^ IC50s of PBI-0451 are mean from *n* = 9 experiments for WT, *n* = 3 for mutant Mpro proteins; for GC376, only *n* = 1 experiment was performed (except for the WT enzyme, showing mean IC50 from *n* = 2 experiments).

In the pomotrelvir inhibition assay, cross-resistance was conferred by many nirmatrelvir resistance substitutions (Table 5). Analysis by resistance level and deconvolution of combinations of substitutions are summarized as follows:

1. No resistance (<threefold). Pomotrelvir was fully active against these 10 substitutions: G15S, T21I, L50F, K90R, T135I, H164N, Q189K, D248E, A260V, and T21I + L50F.
2. Low-level resistance (3- to 10-fold). Found in this group are single substitutions Y54A and S144A. To deconvolute combinations of substitutions, we noticed that while T21I and L50F showed no resistance, double mutants T21I + A173V and L50F + A173V both conferred four to fivefold resistance. Assuming changes at T21 or L50 do not affect distal sites such as A173, we postulate that A173V could confer the same four to fivefold resistance as a single substitution. T21I + T304I, L50F + T304I, and T135I + T304I showed similar levels of resistance (~threefold), suggesting that T304I is likely to confer the same ~threefold resistance as a single substitution. However, the possibility cannot be ruled out that combinations of T21I or L50F with substitutions such as A173V and T304I may act together via yet-unidentified mechanisms; M^pro^ assays with single substitutions A173V and T304I are necessary to fully evaluate the impact of these changes. A173V + T304I conferred 6.7-fold resistance, similar to the more complex combinations of T21I + A173V + T304I (6.1-fold) and T21I + C160F + A173V + V186A + T304I (5.7-fold), demonstrating that these additional amino acid substitutions did not change the resistance level of the double mutant. T21I + L50F + A193*P* + S301P conferred 4.9-fold resistance. Among the substitutions in this quadruple combination, T21I + L50F was shown to confer no resistance, so the observed reduction in pomotrelvir inhibition was likely due to A193P and/or S301P.
3. Moderate-level resistance (10- to 50-fold). Found in this group are single substitutions F140A, E166A, and L167F. The double-mutant L50F + E166A showed similar resistance to the single-substitution E166A (36-fold vs 35-fold); the double-mutant T21I + S144A conferred 13-fold resistance, within twofold of that by the single-mutant S144A (6.7-fold). The results showed that combination with T21I or L50F did not significantly change the resistance of single substitutions. T21I + S144A + T304I conferred 23-fold resistance, approximately multiplicative of resistance by S144A (6.7-fold) and T304I (estimated as ~threefold) as single substitutions, suggesting that S144A and T304I each independently contributed to the resistance of the combined mutant. Of the two combinations containing F140L, F140L + A173V conferred 10-fold resistance and L50F + F140L + L167F + T304I conferred 40-fold resistance. Only data on F140A (14-fold resistance) was available, and testing of the single substitutions F140L is required to deconvolute the aforementioned combinations.
4. High-level resistance (>50-fold). Found in this group are single substitutions E166V and H172Y. E166V conferred 51-fold resistance, and combination of E166V with T21I or L50F did not change its resistance level. The double-mutant H172Y + P252L conferred 62-fold resistance, similar to that conferred by the single-substitution H172Y (71-fold) suggesting that P252L had no impact on pomotrelvir activity in the M^pro^ assay. The triple-mutant L50F + E166A + L167F conferred the highest level of resistance (188-fold), which was approximately multiplicative of the fold increase caused by E166A (36-fold) and L167F (13-fold) as single substitutions, suggesting that E166A and L167F each independently contributed to the resistance of the combined mutant.

The activity of the control M^pro^ inhibitor GC376 (20) was similarly affected by most of these substitutions. One exception was the triple-mutant L50F + E166A + L167F, which conferred 34-fold resistance to GC376 compared with 188-fold to pomotrelvir. There was good correlation of IC50 fold change between GC376 and pomotrelvir (*r* ^2^ = 0.74 by linear regression, after excluding the triple-mutant L50F + E166A + L167F).

To further assess the effect of nirmatrelvir resistance substitutions on pomotrelvir activity, 12 mutations were selected and cloned into SARS-CoV-2-EGFP replicons. Four of the mutant replicons (Y54A, F140A, H164N, and H172Y) generated no or a low number of GFP-positive cells after transfection (Table 6) and were not included in subsequent antiviral assays. Among the eight mutant replicons that were evaluated in the pomotrelvir inhibition assay, pomotrelvir was fully active against T21I, L50F, and T21I + L50F which showed ~fourfold increase in EC50 and EC90; the single substitutions (S144A, E166A, and L167F) conferred a low level of resistance, with EC50 and EC90 changes ranging from 1.9- to 4.4-fold. The combinations of substitutions L50F + E166A and L50F + E166A + L167F showed higher resistance than individual single substitutions, with EC50 fold change of 14- and 48-fold, respectively, and EC90 fold change of 11- and 52-fold, respectively (Table 6).

**TABLE 6:** Replication and pomotrelvir susceptibility of replicons containing nirmatrelvir resistance substitutions

^a^Replicon	EGFP signal		^b^EC50		^b^EC90	
	No. positive cells/well	% of WT	Mean (μM, SD)	Mean fold	Mean (μM, SD)	Mean fold
WT	63	100	0.031 (0.01)	–	0.17 (0.04)	–
E166A	73	116	0.11 (0.02)	3.7	0.71 (0.16)	4.4
F140A	0	0	Not done			
H164N	16	25	Not done			
H172Y	0	0	Not done			
L167F	80	127	0.056 (0.011)	1.9	0.51 (0.21)	3
L50F	93	147	0.014 (0.004)	0.5	0.15 (0.09)	0.9
L50F + E166A	347	549	0.41 (0.06)	14	1.8 (0.4)	11
L50F + E166A + L167F	491	777	1.4 (0.1)	48	8.3 (1.2)	52
S144A	128	202	0.092 (0.022)	3	0.56 (0.03)	3.4
T21I	64	102	0.033 (0.008)	1.1	0.27 (0.1)	1.6
T21I + L50F	46	73	0.11 (0.02)	3.8	0.78 (0.15)	4.7
Y54A	0	0	Not done			

^a^ Mutant replicons are listed in alphabetic order.

^b^ Mean of *n* = 3 experiments.

### Combination studies of pomotrelvir with other antiviral agents

Checkerboard assays (21) were performed to evaluate the inhibitory activity of pomotrelvir in combination with two nucleoside analogs remdesivir and N4 hydroxycytidine (NHC, parent of the prodrug molnupiravir) in A549-hACE2 cells infected with SARS-CoV-2 NLuc. Synergy scores were calculated with SynergyFinder Plus software, which defines that a synergy score between −10 and 10 indicates that the interaction between two drugs is likely to be additive. The results as analyzed by four commonly used synergy analysis methods indicated that the combinations of pomotrelvir with remdesivir and pomotrelvir with NHC were both additive, with synergy scores in the additivity range of −10 to 10 (Table 7).

**TABLE 7:** Synergy scores of pomotrelvir (PBI-0451) in combination with remdesivir or NHC as analyzed by four synergy methods^*a*^

Synergy score by	PBI-0451xRDV	PBI-0451xNHC
ZIP	3.1	0.5
HSA	3.2	3.8
Loewe	7.0	1.7
Bliss	4.8	0.8

^*a*^ RDV, remdesivir; NHC, N4-hyroxycytidine; HSA, highest single agent; ZIP, zero interaction potency.

## DISCUSSION

In this study, we have shown that pomotrelvir is a potent competitive inhibitor of SARS-CoV-2 M^pro^ with high selectivity against host proteases; it is broadly active against SARS-CoV-2 clinical isolates, including Omicron variants. Pomotrelvir is also shown to inhibit other human CoVs in enzyme and cell-based infection assays. Combination studies demonstrate additivity between pomotrelvir and two RdRp inhibitors remdesivir and molnupiravir, which is consistent with the fact that the two classes of inhibitor target different viral proteins (M^pro^ vs viral polymerase), supporting the potential combination with remdesivir and molnupiravir in treating SARS-CoV-2 infections. In contrast, the binding exclusivity study has shown that pomotrelvir and nirmatrelvir to M^pro^ compete for the same binding site, suggesting that co-administration of the two M^pro^ inhibitors is not likely to offer additional improvement in efficacy.

Given the finding that pomotrelvir and nirmatrelvir compete for the same binding site, it is not surprising that many (but not all) nirmatrelvir resistance substitutions confer cross-resistance to pomotrelvir. Of particular interest is the substitution E166V; it was detected in nirmatrelvir/ritonavir-treated patients (14) and conferred 51-fold resistance to pomotrelvir in the M^pro^ assay. However, the resistance profiles of pomotrelvir and nirmatrelvir are not identical. For example, substitutions G15S, T135I, H164N, and D248E were reported by Pfizer to confer ~fourfold resistance in the M^pro^ assay (14) but no change in enzyme IC50 was seen with pomotrelvir in our study, which warrants further comparative studies of resistance variants that may emerge in future SARS-CoV-2 outbreaks. It is notable that all moderate to high-level resistance substitutions (F140A, E166A, E166V, L167F, and H172Y) are associated either with 5- to 10-fold lower M^pro^ catalytic activity (e.g., E166A, E166V, L167F, and H172Y) or with poor replication of mutant SARS-CoV-2 replicons (e.g., F140A and H172Y). This observation suggests that there is a delicate balance between selection of resistance to M^pro^ inhibitors and cost to viral fitness and underlies the potential of developing next-generation M^pro^ compounds which can overcome resistance to current inhibitors like pomotrelvir and nirmatrelvir.

When comparing the results from the mutant M^pro^ enzyme assay with those from the mutant SARS-CoV-2-EGFP replicon assay, we detected two types of discordance. For the double-mutant T21I + L50F (which has fourfold higher catalytic efficiency than WT), no resistance was observed in the M^pro^ assay, but a ~fourfold increase in EC50 and EC90 was seen in the replicon assay. On the contrary, for a series of mutants containing E166A (e.g., E166A, L50F + E166A, and L50F + E166A + L167F) with low enzyme activity, the IC50 fold increase in the M^pro^ assay (36-, 35-, and 188-fold, respectively) is greater than the EC50 fold increase in the replicon assay (3.7-, 14-, and 48-fold, respectively). It is plausible that the M^pro^ enzyme assay is more sensitive in measuring resistance with poorly active enzymes (e.g., E166A), whereas the cell-based replicon assay is better equipped to detect changes in susceptibility caused by the global impact on viral replication such as improved polyprotein processing due to a more active M^pro^ (e.g., T21I + L50 FI). Further studies with additional M^pro^ mutants are required to confirm this observation, but it underscores the importance of conducting both enzymatic and cell-based assays in evaluating the impact of M^pro^ substitutions on SARS-CoV-2 replication and susceptibility to inhibitors.

Results from the *in vitro* characterization of pomotrelvir antiviral activity and phase 1 clinical studies (22) supported its further development and advancement to a phase 2 clinical study in patients who are otherwise healthy with no risk of progression to severe disease (NCT 05543707); the primary endpoint was the effect on the proportion of patients below the limit of detection by infectious virus assay on day 3 of treatment (pomotrelvir vs placebo). Pardes Biosciences recently announced that pomotrelvir did not meet the primary endpoint (https://ir.pardesbio.com/news-releases/news-release-details/pardes-biosciences-announces-top-line-results-phase-2-trial). At this stage of the COVID-19 pandemic, with lower viral burden and more rapid symptom resolution due to high levels of underlying population immunity, it has become more challenging to demonstrate the virologic effect and clinical benefit of SARS-CoV-2 therapeutics on the non-high risk population (i.e., otherwise healthy patients with no risk of progression to severe disease). Other examples of M^pro^ inhibitors in late-stage development include STI-1558, currently in the phase 3 clinical trial in adult subjects with mild or moderate COVID-19 (NCT05716425). Recently, the topline results of the phase 2 trial of the M^pro^ inhibitor EDP-235 have been announced (https://ir.enanta.com/news-releases/news-release-details/enanta-pharmaceuticals-reports-positive-topline-results-phase-2). In similar non-high-risk patients, EDP-235 failed to demonstrate the effect on viral RNA decline or infectious viral load in nasal samples, again highlighting the challenge of demonstrating a virologic effect on this patient population. Despite the progress and challenges, as long as COVID-19 remains as a major public health issue, efforts from public and private sectors should continue to develop safe, highly effective, and orally administered agents without drug-drug interactions for treatment of SARS-CoV-2 infections. Furthermore, a pan-coronavirus inhibitor with high barrier to resistance would be of great value to the preparedness of future coronavirus pandemics. We believe the lessons learned from the development of pomotrelvir will contribute to the understanding of SARS-CoV-2 replication and the discovery of novel therapeutics for COVID-19 and other human CoV infections.

## MATERIALS AND METHODS

### Compounds

Pomotrelvir and nirmatrelvir were synthesized by Pardes Biosciences (Carlsbad, CA). GC376 was provided by WuXi AppTec (Shanghai, China). Compounds were dissolved in DMSO and stored at −20°C.

### Cell lines and viruses

A549-hACE2 cells overexpressing the human ACE2 receptor for SARS-CoV-2 were obtained from Dr. Ralph Baric at UNC Chapel Hill (Chapel Hill, NC) and cultured in Dulbecco’s modified Eagle medium (DMEM) supplemented with 10% fetal bovine serum, 100 U/mL penicillin and streptomycin, and 1× minimal essential amino acids (Corning, Corning, NY). Pluripotent stem cell-derived human lung alveolar type 2 (AT2) cells were cultured as described previously (23) and plated onto 6.5-mm Costar Transwells (Corning) 7–10 d prior to infection until confluency was reached and homogenous expression of the AT2-specific tdTomato reporter was confirmed. Huh7 cells were acquired from WuXi AppTec and maintained in the DMEM supplemented with 10% FBS, 1% L-glutamine, 1% non-essential amino acids (NEAA), and 1% penicillin-streptomycin. MRC5 (ATCC#CCL-171) and Huh7 (JCRB Cell Bank#jcrb0403) cells were obtained from American Type Culture Collection (ATCC) (Manassas, VA) and Japanese Collection of Research Bioresources (JCRB) (Ibaraki city, Japan), respectively. MRC5 cells were maintained in minimal essential media (MEME) obtained from Sigma Aldrich (St. Louis, MO) supplemented with 10% FBS obtained from ExCell Bio (Shanghai, China), 1% L-glutamine, 1% NEAA, and 1% penicillin-streptomycin (HyClone, Logan, UT). All tissue culture reagents were purchased from Gibco Life Technologies (Grand Island, NY) unless indicated otherwise.

A passage 3 stock of the SARS-CoV-2 WA-1 isolate (MN985325) was obtained from the CDC (Atlanta, GA) and passed twice in Vero E6 cells to generate high-titer passage 5 stock for experiments described in this study. SARS-CoV-2-expressing nanoluciferase (SARS-CoV-2 NLuc) was generated by cloning the seven genomic fragments from the clinical isolate WA1 separately into vector plasmids. The nanoluciferase gene was introduced into the ORF7 (24, 25). The SARS-CoV-2-EGFP replicon was constructed by WuXi AppTec. The replicon backbone was derived from SARS-CoV-2 Wuhan-Hu-1 (NC045512.2) and contained the EGFP reporter gene. To construct replicons containing substitutions in the M^pro^ (nsp5) sequence, mutations were designed by SnapGene software obtained from GSL Biotech, LLC (Boston, MA), and introduced into the plasmid containing the SARS-CoV-2 WT replicon using the two-step red-mediated recombination method (26). The cloned mutations were verified by Sanger sequencing. Mutant replicon RNA was generated by the mMACHINE T7 Ultra Kit (lnvitrogen, Carlsbad, CA) and used to transfect Huh7 cells. One day after transfection, the RNA of transfected cells was extracted, reverse transcribed into cDNA, and subjected to sequencing to verify that the mutant viral RNA that replicated post-transfection maintained the appropriate substitution(s). CoV-229E (ATCC#VR-740) and CoV-OC43 (ATCC#VR-1558) strains were obtained from ATCC. All work at Vanderbilt University Medical Center using SARS-CoV-2 was reviewed and approved by the Institutional Biosafety Committee under BSL-3 protocol registration ID VBMR-0132.

### Continuous kinetic M^pro^ assay

The assay was performed by Nanosyn Inc. (Santa Clara, CA). To determine maximal velocity (*V*_max_), Michaelis constant (*K*_m_), and inhibition constant (*K*_i_), pomotrelvir was tested in the presence of 3 nM SARS-CoV-2 M^pro^ (BPS Biosciences, San Diego, CA) and 2.3–300 μM (1:2 dilution starting at 300 µM) of substrate peptide (FAM-TSAVLQSGFRK-NH2) in assay buffer (20 mM Tris-HCl pH7.3, 100 mM NaCl, 0.1% BSA, 1 mM DTT) at room temperature; cleavage was measured by microfluidic electrophoresis using Caliper’s LabChip 3000 (Calipher Life Sciences, Ind., Hopkinton, MA). Initial velocity (*v*) was derived from progress curves, *V*_max_, and *K*_m_ were determined using the Michaelis-Menten model. To determine the inhibition constant *K*_i_, a global fit was performed using the following general mixed inhibition model:


$$fit=((V_{max}{}^{*}[S])/((K_{m}{}^{*}(1+(l/K_{i})))+[S{]}^{*}(1+([I]/(Alpha^{*}K_{i}))),$$

in which [*I*] is the concentration of compound and Alpha is the parameter describing deviation from pure competitive, non-competitive, or un-competitive models. When Alpha >> 1, the inhibitor preferentially binds to free enzyme. When alpha < 1, the inhibitor preferentially binds to the enzyme-substrate complex. When Alpha = 1, the inhibitor binds to free enzyme and the enzyme-substrate complex with equal affinity. Because the returned *K*_i_ value (5.2 nM) was very close to enzyme concentration (3 nM), Morrison correction was made to account for compound depletion using the following equation:


$$K_{iapp}=(([S]+K_{m})/((K_{m}/K_{i})+([S]/({Alpha}^{*}K_{i})))).$$

The final analysis returned a *K*_i_ of 2.7 nM. All local curve fittings and global fit were performed by using XLFit software (IDBS, Boston, MA).

To evaluate the binding exclusivity between pomotrelvir and nirmatrelvir, initial velocity was determined in the presence of pomotrelvir (0–20 nM) and nirmatrelvir (0–8 nM) with 3 nM enzyme and 1 µM peptide substrate in the assay buffer described above. The binding mode was assessed by the Yonetani-Theorell equation:


$$1/V_{12}=1/V{{}_{0}}^{*}(1+[I_{1}]/(K_{i1}+[I_{2}]/K_{i2}+([I_{1}{]}^{*}[I_{2}])/g(K_{i1}{}^{*}K_{i2})),$$

where *V*_12_ is the initial velocity in the presence of the two test compounds, *V*_o_ is the initial velocity in the control sample (without compounds), *I*_1_ and *I*_2_ are concentrations of the two test compounds, *K*_i1_ and *K*_i2_ are constants of interaction of the two test compounds with M^pro^ enzyme, and *γ* is the binding exclusivity coefficient. If *γ* = 1, the two compounds bind independently of one another to two different binding sites; for compounds that positively affect each other’s binding, *γ* < 1. If *γ* has a finite value > 1, the two compounds demonstrate antagonistic binding; a finite but large (>>1 to ∞) value of *γ* indicates that the two compounds bind to the same site on the enzyme.

Evaluation of pomotrelvir inhibition of host proteases in the kinetic assay was performed by Nanosyn Inc. following protocols established by the facility.

### Endpoint assay for IC50 determination of M^pro^ enzymes

The assay was performed by WuXi AppTec (Shanghai, China). To generate WT SARS-CoV-2 M^pro^, codon-optimized cDNA of SARS-CoV-2 M^pro^ (QIZ13716.1_ORF1a) was cloned into the pET28a vector, expressed in *E. coli*, and purified. To generate mutant SARS-CoV-2 M^pro^ proteins, cDNAs containing resistance substitution(s) were synthesized and cloned into the pET28a vector. The reaction buffer consisted of 20 mM of Tris-HCl (pH7.3), 100 mM of NaCl, 1 mM of EDTA, 5 mM of TCEP, and 0.1% BSA. The cleavage of substrate (Dabcyl- KTSAVLQ‖SGFRKM-Edans) obtained from Genscript (Piscataway, NJ) was measured at 30°C by a SpectraMax M2e microplate reader (Molecular Devices, Silicon Valley, CA). The kinetic parameters (*K*_m_, *k*_cat_) of each mutant protease were determined using the Michaelis-Menten model. The enzyme and substrate concentration and reaction time were selected for each mutant M^pro^ to ensure that the assay was within the linear range, the signal-to-noise ratio (S/N) was ≥3, and the substrate concentration was one- to twofold greater than *K*_m_.

M^pro^ proteins from other human CoVs were also cloned, expressed in *E. coli* and purified by WuXi AppTec. The accession numbers and sequences of peptide substrates are shown as follows: CoV-229E_M^pro^ (AGW80947.1), YGSTLQAGLRKM; CoV-OC43_M^pro^ (AGT51517.1), KTSAVLQSGFRKM; SARS_M^pro^ (AAR87511.1), KTSAVLQSGFRKM; MERS_M^pro^ (ASU89965.1), KTSAVLQSGFRKM; CoV-NL63_M^pro^ (AGT51378.1), YNSTLQSGLKKM; and CoV-HKU1_M^pro^ (ARB07597.1), KTSAVLQSGFRKM. The assay conditions were similar to those of SARS-CoV-2 M^pro^.

### Co-crystallization and structure determination

The study was performed by Schrödinger Inc. (Natick, MA). The SARS-CoV-2 Mpro protein provided by WuXi AppTec was diluted to 7 mg/mL and mixed with pomotrelvir at a 2:1 molar ratio in 20 mM Hepes pH 7.5, 50 mM NaCl and 2.5 mM DMSO and incubated for 60 min at 4°C. The sample was then clarified by centrifugation at 16,800 × *g* for 10 min at 10°C and set up for co-crystallization using the sitting drop method against reservoir containing 24.1% PEG 3350, 0.1 MES pH 7.5 and incubated at 20°C.

Co-crystals were harvested and flash frozen in liquid nitrogen directly from the crystallization drop. X-ray diffraction data were collected at beamline BL13-XALOC, ALBA synchrotron, Cerdanyola del Vallès, Barcelona, Spain, using a wavelength of 0.979261 Å. The X-ray diffraction data were reduced using XDS (27), scaled, and averaged using AIMLESS (CCP4 suite). The structure was solved by molecular replacement using PHASER and the RCSB entry 6YB7.pdb where the ligand, waters, and other solvents were excluded from the model. Model building and refinement were performed using COOT (28) and REFMAC5 (29) with TLS refinement, respectively (Fig. 1C). The resulting structure has been deposited with RCSB as entry 8TBE.pdb.

### Evaluation of pomotrelvir activity in human lung alveolar type 2 cells

AT2 cells were infected with SARS-CoV-2 WA-1 at a multiplicity of infection (MOI) of 0.004 plaque-forming units (PFU)/cell in AT2 medium (23) for l hr at 37°C. Viral inoculum was removed, and cells were washed three times with basal medium before addition of testing compounds. At 48 hr post infection, 150–200-μL medium was added to the apical compartment for 10 min and removed and the apical wash was processed for virus quantification by plaque assay and RT-qPCR. To quantitate infectious viral titer by plaque assay, Vero E6 cells were incubated with 10-fold serial dilutions of virus-containing supernatants in gel saline which were adsorbed in duplicates for 30 min at 37°C. Cells were then overlaid with a 1:1 mixture of 2× DMEM and 2% agar in ddH2O and incubated at 37°C for 72 hr. Plaques were enumerated in unstained monolayers using a light box. To quantitate the viral RNA copy number, virus-containing supernatants were treated with TRIzol LS Reagent (Invitrogen, Waltham, MA), and RNA was purified following phase separation by chloroform as recommended by the manufacturer. RNA in the aqueous phase was collected and further purified using Purelink RNA Mini Kits (Invitrogen) according to the manufacturer’s protocol. Viral RNA was quantified by RT-qPCR on a StepOnePlus Real-Time PCR System (Applied Biosystems, Bedford, MA) using TaqMan Fast Virus 1-Step Master Mix Chemistry (Applied Biosystems). Primers for SARS-CoV-2 nsp4 gene RNA were obtained from Sigma Aldrich and amplified using forward (5′-GTGCTCATGGATGGCTCTATTA-3′), reverse (5′CGTGCCTACAGTACTCAGMTC-3′) primers, and probe (5′-FAMACCTACCTTGMGGTTCTGTTAGAGTGGT-BHQl-3′). RNA copy numbers were interpolated from a standard curve produced with serial 10-fold dilutions of nsp4 gene RNA run on the same sample plate. Cell viability was determined by the Toxilight Bioassay Kit from Lonza (Basel, Switzerland) according to the manufacturer’s instructions. Luminescence was detected using a Synergy Hl Plate Reader (BioTek, Winooski, Vermont).

### SARS-CoV-2 Nluc assay in A549-hACE2 cells

A549-hACE2 cells were infected with SARS-CoV-2 NLuc at an MOI of 0.025. After incubation for 45 min, inoculum was removed, and cells were treated with titrations of pomotrelvir for 48 hr in growth media containing 10% FBS supplemented with 0, 5, 10, or 20% human serum (MP Biomedicals, Solon, OH). Viral replication was quantified by measuring nanoluciferase activity using the Nano-Glo Luciferase Assay System (Promega, Madison, WI) according to the manufacturer’s specifications. Cell viability was determined by CellTiter-Glo Assay (Promega) according to the manufacturer’s instructions. Luminescence was detected using a Synergy H1 Plate Reader (BioTek).

### SARS-CoV-2 replicon assay

The assay was performed by WuXi AppTec. Huh7 cells were transfected with *in vitro* transcribed replicon RNA and seeded at 4,000 cells/well in 384-well plates containing serially diluted compounds and then cultured at 37°C for 1 d. The replication of SARS-CoV-2 replicon was measured by the expression of the EGFP reporter gene. Fluorescence intensity was determined using Acumen Cellista (TTP LabTech, Melbourn, UK), and the antiviral activity of compounds was calculated based on the reduction of number of GFP-positive cells. The acceptable threshold of GFP signal was >30 GFP-positive cells/well. Cell viability was measured with CellTiter-Glo following manufacturer’s instructions.

### Pomotrelvir inhibition of clinical isolates

The assay was performed by Retrovirox Inc. (San Diego, CA). A549 cells overexpressing ACE2 and TMPRSS2 (Retrovirox Inc.) were plated in DMEM with 10% FBS for 24 hr followed by addition and incubation of serial dilutions of test compounds (in DMED with 2% FBS) for 1 hr at 37°C. Cultures were then infected with SARS-CoV-2 resuspended in DMEM with 2% FBS at MOI of 0.005 TCID50/cell. Cell culture media with the virus inoculum was not removed after virus adsorption. After 48 hr of incubation, the extent of infection was monitored by immunostaining using a monoclonal antibody against the SARS-CoV-2 nucleocapsid (NP) followed by incubation with horseradish peroxidase conjugated polyclonal antibodies against human IgG (HRP-donkey anti-human IgG). The expression of the viral antigen (NP) in infected cells were quantitated using a colorimetric readout (absorbance at 492 nm). Cell viability was determined with the CellTiter 96 AQueous One Solution cell proliferation assay (MTS) from Promega. The clinical isolates used in the study are listed as follows:

MEX-BC2/2020, representative of the B.1 lineage, carrying the D614G mutation (GISAID database ID: EPI_ISL_747242), MEX-BC12/2021, representative of the B.1.1.7 lineage, referred as the “Alpha” variant of concern (GISAID database ID: EPI_ISL_2455241), MEX-BC10/2021, representative of the B.1.429 lineage referred as the “Epsilon” variant (GISAID database ID: EPI_ISL_2455244), MEX-BC15/2021, representative of the B.1.617.2 lineage, referred as the “Delta” variant of concern (GISAID database ID: EPI_ISL_2249247), MEX-BC18/2021, representative of the B.1.617.2 + AY.20 lineage, also referred to as the “AY.20 Delta” variant (GISAID database ID: EPI_ISL_9149176), MEX-BC16/2021, representative of the B.1.621 lineage, referred to as the “Mu” variant (GISAID database ID: EPI_ISL_6944671), RVX-01/2022, representative of the BA.1.1 sub-lineage of the Omicron variant of concern (GISAID database ID: EPI_ISL_10954105), USA/RVX05/2022, Omicron BA.2 sub-lineage (EPI_ISL_12470781), USA/RVX07/2022, representative of BA.2.12.1 sub-lineage of the Omicron variant of concern (EPI_ISL_13351626), MEX/BC28/2022, Omicron BA.4.1 sub-lineage (EPI_ISL_14319831), USA/RVX13/2022, Omicron BA.5.2.1 sub-lineage (EPI_ISL_14319830).

### Inhibition of CoV-229E and CoV-OC43 in cell-based CPE assays

In the CoV-229E assay, compounds diluted in assay medium (MEME supplemented with 5% FBS, 1% L-glutamine, 1% NEAA, and 1% penicillin-streptomycin) were added to MRC5 cells (seeded at 20,000 per 96-well for overnight), which were then infected with 200 TCID50 per well of CoV-229E virus. The resulting cultures were incubated at 35°C and 5% CO2 for an additional 3 d, at which time the virus infection in the virus control (cells infected with virus, without compound treatment) displayed significant CPE. In the CoV-OC43 assay, compounds diluted in cell culture medium were added to Huh7 cells (seeded at 8,000 per 96-well for overnight), which were then infected with 100 TCID50 per well of CoV-OC43 virus. The resulting cultures were incubated at 33°C and 5% CO2 for an additional 7 days, at which time the virus infection in the virus control displayed significant CPE. The CPE was measured by CellTiter-Glo following the manufacturer’s manual.

### Data analysis of endpoint M^pro^ and cell-based activity assays

IC50, EC50, EC90, and CC50 values were calculated using the nonlinear regression model of log(inhibitor) vs response-variable slope (four parameters) by Graphpad Prism (San Diego, CA) or by Dotmatics software (Boston, MA). IC50 and EC50 were defined as the drug concentration achieving the response halfway between the fitted bottom and top (maximum) inhibition%; EC90 was defined as the drug concentration achieving 90% inhibition between the fitted bottom and top (maximum) inhibition%. To calculate fold change, the activity (e.g., IC50, EC50, and EC90) of each mutant was divided by the activity of the corresponding WT from the same experiment.

### Combination study of pomotrelvir with remdesivir and NHC

A549-hACE2 cells were infected with SARS-CoV-2 NLuc at an MOI of 0.025 PFU/cell and incubated for 48 hr with 0.0013–1 μM pomotrelvir in combination with 0.02–2.5 μM remdesivir (RDV) or 0.0013–3 μM of pomotrelvir in combination with 0.04–2.5 μM NHC. Viral replication was quantified by measuring nanoluciferase activity using the Nano-Glo Luciferase Assay System (Promega). To carry out synergy analysis, the nanoluciferase signal expressed from SARS-CoV-2 NLuc infection treated with pomotrelvir × RDV or pomotrelvir × NHC was normalized to signals from cells treated with DMSO alone and percent inhibition was calculated. The resulting data sets served as input in SynergyFinderPlus software (https://synergyfinder.org/#!/) to calculate ZIP synergy scores (30). When the overall summary synergy score is (https://synergyfinder.fimm.fi/synergy/synfin_docs/#datanal)

- Less than −10, the interaction between two drugs is likely to be antagonistic.
- From −10 to 10, the interaction between two drugs is likely to be additive.
- Larger than 10, the interaction between two drugs is likely to be synergistic.
